# Analysis of Suspected Measles Cases with Discrepant Measles-Specific IgM and rRT-PCR Test Results, Japan

**DOI:** 10.3201/eid3005.231757

**Published:** 2024-05

**Authors:** Yumani Kuba, Minoru Nidaira, Noriyuki Maeshiro, Katsuhiro Komase, Hajime Kamiya, Hisako Kyan

**Affiliations:** Okinawa Prefectural Institute of Health and Environment, Okinawa, Japan (Y. Kuba, M. Nidaira, N. Maeshiro, H. Kyan);; National Institute of Infectious Diseases, Tokyo, Japan (K. Komase, H. Kamiya)

**Keywords:** Measles, viruses, vaccine-preventable diseases, IgM testing, IgG avidity, febrile exanthematous viruses, Japan

## Abstract

We investigated clinically suspected measles cases that had discrepant real-time reverse transcription PCR (rRT-PCR) and measles-specific IgM test results to determine diagnoses. We performed rRT-PCR and measles-specific IgM testing on samples from 541 suspected measles cases. Of the 24 IgM-positive and rRT-PCR­–negative cases, 20 were among children who received a measles-containing vaccine within the previous 6 months; most had low IgG relative avidity indexes (RAIs). The other 4 cases were among adults who had an unknown previous measles history, unknown vaccination status, and high RAIs. We detected viral nucleic acid for viruses other than measles in 15 (62.5%) of the 24 cases with discrepant rRT-PCR and IgM test results. Measles vaccination, measles history, and contact history should be considered in suspected measles cases with discrepant rRT-PCR and IgM test results. If in doubt, measles IgG avidity and PCR testing for other febrile exanthematous viruses can help confirm or refute the diagnosis.

Measles is a highly contagious febrile exanthematous disease caused by the measles virus. The measles virus spreads via airborne and droplet transmission and can cause severe complications, such as pneumonia, acute encephalitis, and sometimes death (*1*). Vaccination with 2 doses of measles-containing vaccine (MCV) is the best way to protect against measles virus infection and achieving and maintaining a high level of immunity in a population can prevent the spread of the virus (*2*).

In March 2015, the World Health Organization Western Pacific Regional Office verified Japan as a country having achieved measles elimination (*3*). However, although measles frequency has decreased since the achievement of elimination (*4*), outbreaks initiated by measles-susceptible persons traveling to or from measles-endemic countries still occur (*5*–*7*). Therefore, enhanced measles surveillance has been ongoing in Japan since achieving elimination status. 

Samples from clinically suspected measles cases are required to undergo laboratory testing. Although ELISA detection of specific measles virus IgM in serum is the standard diagnostic method for measles (*8*), detection of measles virus RNA in clinical specimens via real-time reverse transcription PCR (rRT-PCR) is considered the most reliable diagnostic test during the first few days after rash onset (*9*). In Japan, recommendations call for taking 3 specimens (throat swab, and blood and urine samples) from patients with clinically suspected measles and performing rRT-PCR testing to detect measles viral RNA in addition to measles-specific IgM testing (*10*). Samples collected from 3 days before the onset of the fever or rash symptoms to 1 week after the onset of the rash are appropriate for rRT-PCR testing (*10*).

In Okinawa Prefecture, Japan, no confirmed measles case had been reported since 2014, then a prefecture-wide measles outbreak occurred during March–May 2018 (*7*). Samples were collected from all persons suspected of having measles and were subjected rRT-PCR and IgM testing at the Okinawa Prefectural Institute of Environment and Health (OPIEH). For most cases, laboratory testing confirmed the diagnosis, but for some cases, rRT-PCR and IgM test results were inconsistent, IgM-positive and rRT-PCR–negative results. Because the public health response differs depending on whether measles is confirmed, we conducted additional laboratory testing in conjunction with collecting additional patient epidemiologic information to make an accurate diagnosis of measles in cases with discrepant IgM and rRT-PCR results.

## Material and Methods

All specimens collected from persons with suspected measles in Okinawa during the 2018 outbreak underwent rRT-PCR testing to confirm the diagnosis. IgM testing was also performed for all cases with serum samples. If the specimen collection period was appropriate, the rRT-PCR–positive result was defined as a confirmed measles case regardless of the measles-specific IgM test result. An rRT-PCR–negative and IgM-negative or IgM-equivocal result was also defined as a non–measles case. For cases with rRT-PCR–negative and IgM-positive results, we could not determine a diagnosis because of the inconsistency of the 2 test results. For those cases, we collected demographic information, including vaccination and exposure histories, to evaluate the test discrepancies. Furthermore, we conducted measles IgG avidity testing by using serum samples and an rRT-PCR or conventional PCR by using throat swab, serum, and urine samples to detect other viruses that cause fever and exanthemata. We chose target viruses that cause febrile exanthematous illnesses and can cause cross-reactions with the measles-specific IgM tests on the basis of reports from previous studies (*11*). Target viruses included rubella virus, human herpesvirus 6 (HHV-6), human herpesvirus 7 (HHV-7), parvovirus B19 (B19), Epstein-Barr virus (EBV), cytomegalovirus (CMV), human parechovirus (HPeV), enterovirus, and adenovirus. This study was approved by the ethics committee of the Okinawa Prefectural Institute of Health and Environment (approval no. 694-2).

### Data Collection

Under the Infectious Disease Control Law in Japan (*12*), all 6 public health centers in Okinawa Prefecture are required to collect information on suspected measles cases, including demographic characteristics, symptoms, onset date, vaccination history, and outcomes. Those data were sent to the OPIEH and used for the analysis.

### Specimen Collection and Pretreatment

Physicians collected throat swab, whole blood, and urine samples from persons with suspected measles and local public health center staff delivered samples to the OPIEH under refrigerated conditions. OPIEH performed measles-specific rRT-PCR testing by extracting viral nucleic acid from 140 µL of each specimen by using the QIAmp Viral RNA Mini Kit (QIAGEN, https://www.qiagen.com). Staff isolated serum from blood and tested serum for measles-specific IgM and IgG and by using IgG avidity tests.

### Measles-Specific rRT-PCR

We performed rRT-PCR as reported previously (*7*). We used MVN1139F (5′-TGGCATCTGAACTCGGTATCAC-3′) and MVN1213R (5′-TGTCCTCAGTAGTATGCATTGCAA-3′) primers and an MVNP1163P probe (5′-FAM-CCGAGGATGCAAGGCTTGTTTCAGA-TAMRA-3′) targeting the nucleocapsid (N) gene of measles virus (*13*).

### Measles-Specific IgM

We tested serum samples for measles-specific IgM by using the Measles IgM-EIA (Denka Seiken, Ltd., https://denka-seiken.com), an IgM capture assay. We interpreted test results in accordance with the manufacturer’s definition: positive, >1.2 relative unit (RU); equivocal, 0.8–1.2 RU; and negative, <0.8 RU.

### Measles-Specific IgG and IgG Avidity Tests

We used the Anti-Measles Virus ELISA (IgG) (EUROIMMUN, https://www.euroimmun.com) to detect measles-specific IgG. We interpreted results in accordance with the manufacturer’s definitions: positive, >275 IU/L; borderline, >200 to <275 IU/L; and negative, <200 IU/L.

We measured measles-specific IgG avidity by using the Anti-Measles Virus IgG Avidity ELISA Kit (EUROIMMUN). We calculated the relative avidity index (RAI) for each sample according to the manufacturer’s instructions: RAI <40% indicated low avidity antibodies, RAI 40%–60% equivocal, and RAI >60% indicated high avidity antibodies. IgG avidity test results can help distinguish recent primary infection, characterized by a low RAI, from past infection, characterized by a high RAI (*14*,*15*).

### Febrile Exanthematous Virus Detection

For rRT-PCR­–negative but IgM-positive samples, we extracted viral nucleic acid and used PCR and rRT-PCR to test for 9 different viruses: rubella virus, HHV-6, HHV-7, B19, EBV, CMV, HPeV, enterovirus, and adenovirus (*16*–*24*). Those viruses are known to cause febrile exanthemata and to cross-react with measles-specific IgM (*11*). Previous reports showing that the QIAmp VIral RNA Mini Kit (QIAGEN) effectively isolates viral DNA (*25*,*26*). Thus, we used that kit to extract viral nucleic acid for detecting DNA and RNA viruses using various primers and probes (Appendix Table 1). We used Ex Taq DNA Polymerase (TaKaRa Bio, Inc., http://www.takara-bio.com) for PCR testing to detect EBV and CMV under the following conditions: 10 minutes at 95°C, followed by 10 cycles for 30 seconds at 95°C, 30 seconds at 70–61°C with a 1°C decrease in temperature per cycle, and 1 minute at 72°C, followed by 35 cycles for 30 seconds at 95°C, 30 seconds at 60°C, and 30 seconds at 60°C. Finally, we performed an additional extension step for 5 minutes at 72°C. We performed the PCR test to detect adenovirus under the following conditions: 3 min at 94°C, followed by 40 cycles for 30 seconds at 94°C, 60 seconds at 50°C, 2 minutes at 72°C, and 5 minutes at 72°C. We performed the rRT-PCR test by using 4 × TaqMan Fast Virus 1-step Master Mix (Thermo Fisher Scientific, https://www.thermofisher.com) under the following conditions: 5 minutes at 50°C, 20 seconds at 95°C, followed by 40 cycles of 15 seconds at 95°C and 1 minute at 60°C. We validated the sensitivity of the test by confirming that serially diluted virus-positive control RNA or DNA was detectable up to ≈5–50 copies per reaction. We included a negative control (no viral genome) and a positive control (viral RNA or DNA) in each test. 

## Results

**Figure 1:**
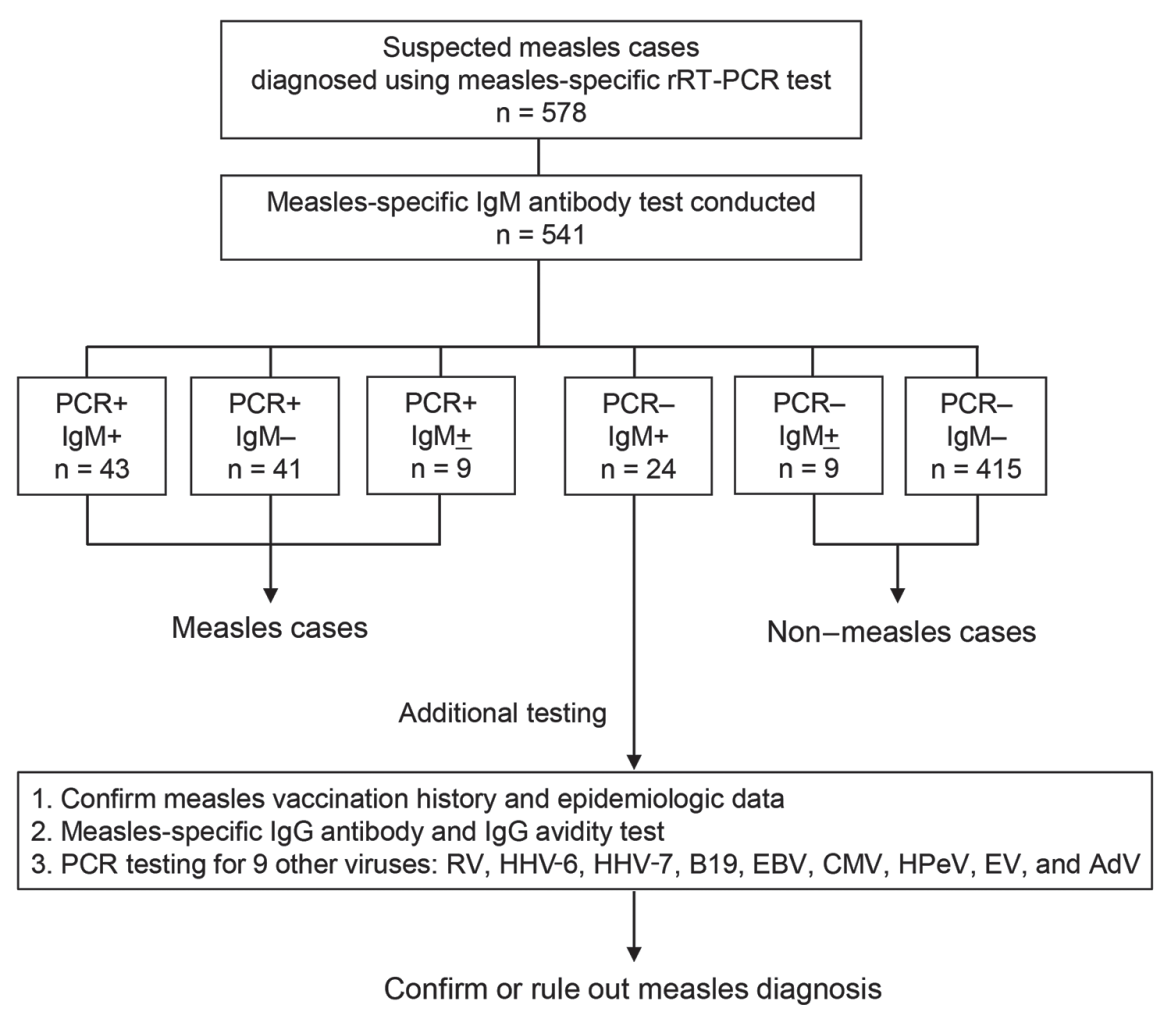
Flow diagram for analysis of suspected measles cases with discrepant measles-specific IgM and rRT-PCR test results, Japan. AdV, adenovirus; B19, parvovirus B19; CMV, cytomegalovirus; EBV, Epstein-Barr virus; EV, enterovirus; HHV-6, human herpesvirus 6; HHV-7, human herpesvirus 7; HPeV, human parechovirus; rRT-PCR, real-time reverse transcription PCR; RV, rubella virus; + positive, ­–, negative; +, equivocal.

We conducted rRT-PCR testing on samples from 578 persons with suspected measles, of which samples from 541 (93.6%) persons also underwent serologic testing (Figure 1). Of those 541 suspected cases, 93 (17.2%) were diagnosed as measles on the basis of rRT-PCR using specimens collected within 7 days of symptom onset. Among the other 448 (82.8%) specimens, 424 were collected during the appropriate period and were classified as non–measles cases on the basis of rRT-PCR and IgM test results. However, 24 of the 448 rRT-PCR–negative cases tested positive for measles-specific IgM, resulting in discrepant rRT-PCR and IgM test results (Figure 1). For those 24 cases, we collected vaccination history and epidemiologic information, such as history of contact with confirmed cases or viral transmission to others, to support the diagnosis. Furthermore, we performed additional measles IgG, IgG avidity, and detection of other febrile exanthematous viruses to support the diagnoses.

We collected characteristics of the 24 patients with discrepant laboratory test results (Table 1). Of those patients, 19 (79.2%) were infants <1 year of age, 1 (4.2%) was child 4 years of age, and 4 (16.7%) were adults >20 years of age. All 20 children had a history of receiving a dose of MCV within 6 months before specimen collection, and 17 (85%) were vaccinated within 60 days before specimen collection. The vaccination histories of the 4 adult patients were unknown. Of note, 12 infants 6–11 months of age received MCV because the Okinawa prefectural government made infants in that age range eligible for MCV vaccination as an emergency response to measles outbreak (*7*). None of the patients with inconsistent test results had epidemiologic links to laboratory-confirmed measles cases. Moreover, we observed no secondary measles cases associated with those cases. Specimens were collected within 10 days after the fever onset, and the median measles-specific IgM result was 2.32 (range 1.23–6.83) RU (Table 2).

**Table 1:** Characteristics of 24 suspected measles cases with discrepant measles-specific IgM and rRT-PCR test results, Japan*

Characteristics	Value
Sex	
M	13 (54.2)
F	11 (45.8)
Age	
6–11 mo	12 (50.0)
1 y	7 (29.2)
4 y	1 (4.2)
>19 y	4 (16.7)
Fever, temperature >37.5°C†	20 (83.3)
Rash	23 (95.8)
No. doses of measles vaccine‡	
1	20 (83.3)
2	0 (0)
Unknown§	4 (16.7)
Median time from vaccination to specimen collection, d (range)	25 (4–136)
Time from illness onset to specimen collection, d	
Median (range)	3 (0–10)
<4	14 (58.3)
>4	10 (41.7)
No epidemiologic link to confirmed measles case	24 (100)

*Values are no. (%) except as indicated. rRT-PCR, real-time reverse transcription PCR. †The other 4 cases also had reported fever, but details of body temperature were unknown. ‡In Japan, the measles-rubella vaccine is used for routine vaccination and has the following schedule: first dose at 1 year of age, second dose at 5–7 years of age. During the 2018 measles outbreak in Okinawa, Japan, 12 infants 6–11 months of age were vaccinated as part of the outbreak response before routine vaccination. **§Four adult cases had no details of vaccination history. Among those cases, 1 patient recalled receiving 2 doses of the measles vaccine but did not have a vaccination record.**

**Table 2:** Characteristics and laboratory results for 24 suspected measles cases with discrepant measles-specific IgM and rRT-PCR test results, Japan*

Case no.	Age/ sex	No. measles vaccine doses	Fever, °C	Rash	Time to specimen collection, d		IgM/ IgG†	% RAI, avidity‡	Other febrile exanthematous viruses detected on PCR			Results§	Measles virus infection
					After vaccine	After fever onset			Throat swab	Serum	Urine		
1	6 mo/M	1	39.5	Y	4	3	1.37/ND	ND, L	HHV-6	HHV-6	ND	CX or MCV	N
2	1 y/F	1	40.0	Y	6	0	1.56/ND	ND, L	CMV	ND	ND	CX or MCV	N
3	1 y/M	1	40.0	Y	18	2	4.71/ 984.9	16.6, L	HHV-6	HHV-6	ND	MCV	N
4	4 y/F	1	+	N	26	1	1.78/ 2,286	35.9, L	EBV, HHV-6, CMV	ND	CMV	MCV	N
5	10 mo/M	1	37.9	Y	11	1	1.74/ 149.2	39.6, L	CMV	ND	CMV	CX or MCV	N
6	9 mo/M	1	38.0	Y	16	0	3.96/ 1,200	21.7, L	HHV-6, CMV	ND	ND	MCV	N
7	11 mo/F	1	Y	Y	21	1	6.83/ 1,242	24.6, L	CMV	ND	CMV	MCV	N
8	6 mo/M	1	38.7	Y	16	0	5.54/ 1,224	15.6, L	CMV	HHV-6, CMV	HPeV, CMV	MCV	N
9	7 mo/F	1	39.0	Y	12	2	2.74/ 14.4	17.3, L	ND	ND	ND	MCV	N
10	9 mo/M	1	38.3	Y	24	0	2.53/ 1,037	14.0, L	ND	ND	ND	MCV	N
11	7 mo/M	1	38.2	Y	42	0	2.50/ 2,112	17.5, L	ND	HPeV	ND	MCV	N
12	11 mo/M	1	39.1	Y	58	1	2.39/ 1,033	18.4, L	ND	ND	ND	MCV	N
13	1 y/F	1	39.3	Y	47	6	1.23/ 1,606	27.9, L	ND	CMV	CMV	MCV	N
14	7 mo/F	1	38.0	Y	25	4	2.15/ 396.7	12.3, L	ND	ND	ND	MCV	N
15	6 mo/F	1	38.9	Y	18	10	4.56/ 1,118	17.8, L	ND	ND	ND	MCV	N
16	1 y/M	1	Y	Y	33	5	1.42/ 1,047	28.1, L	HHV-6, HPeV	HHV-6	ND	MCV	N
17	10 mo/F	1	39.4	Y	41	4	1.37/ 4,440	25.5, L	HHV-6	HHV-6	HHV-6	MCV	N
18	1 y/M	1	40.4	Y	61	6	3.82/ 1,825	47.9, E	ND	ND	ND	MCV	N
19	1 y/F	1	39.0	Y	88	3	1.45/ >5,000	50.6, E	ND	ND	ND	MCV	N
20	1 y/F	1	40.6	Y	136	10	2.25/ 2,076	76.1, H	ND	HHV-6	ND	MCV or CX	N
21	24 y/F	Unk	38.8	Y	Unk	2	2.55/ >5,000	79.7, H	HHV-7	ND	ND	RI or CX	Y or N
22	21 y/M	2	38.0	Y	Unk	5	2.05/ >5,000	88.4, H	ND	ND	ND	RI	Y
23	29 y/M	Unk	Y	Y	Unk	5	3.89/ 4,228	91.7, H	ND	ND	ND	RI	Y
24	45 y/M	Unk	39.1	Y	Unk	5	1.29/ 518.7	91.0, H	HHV-7, B19	B19	ND	RI or CX	Y or N

*B19, human parvovirus B19; CMV, cytomegalovirus; CX, cross-reaction; E, equivocal; EBV, Epstein-Barr virus; H, high; HHV-6, human herpesvirus 6; HHV-7, human herpesvirus 7; HPeV, human parechovirus; L, low; MCV, measles-containing vaccine; ND, not detected; RAI, relative avidity index; RI, reinfection; Unk, unknown. †IgM values indicate RU; IgG values indicate IU/L. ‡IgG avidity test results can help distinguish recent primary infection (characterized by a low RAI) from past infection (characterized by a high RAI). RAI <40% indicated low-avidity antibodies, RAI 40%–60% equivocal, and RAI >60% indicated high-avidity antibodies. §MCV indicates that an increased IgM titer might be influenced by vaccination with a measles-containing vaccine; CX indicates a cross-reaction of a febrile exanthematous virus infection other than the measles virus, respectively.

We measured measles-specific IgG titer and conducted measles IgG avidity testing on samples from the 24 persons with measles-specific IgM-positive and rRT-PCR­–negative results. We used results from those tests to distinguish between a recent primary infection and previous contact with either wild-type measles virus or vaccination as the cause of positive measles-specific IgM results. Among the 20 children, 16 had positive measles-specific IgG results (range 396.7 to >5,000 IU/L). The 4 (cases 1, 2, 5, and 9) children who had negative measles-specific IgG results had their first MCV vaccination within 2 weeks before specimen collection (Table 2). Although all 4 adult cases had positive measles-specific IgG results, a 45-year-old patient (case 24) had a relatively low IgG titer (518.7 IU/L) compared with the other 3 adults (>4,000 IU/L) who were all in their 20s (Table 2).

Among the 20 children, 17 had a low RAI, and 3 children (cases 18–20) had equivocal or high RAIs. The median interval from vaccination to specimen collection was 25 (range 4–136) days. The RAIs of the 20 children who had received 1 dose of MCV vaccine correlated with the number of days since vaccination (R^2^ = 0.6877) and tended to increase over time after vaccination (Appendix Figure 1). All 4 adult cases had high RAIs.

**Figure 2:**
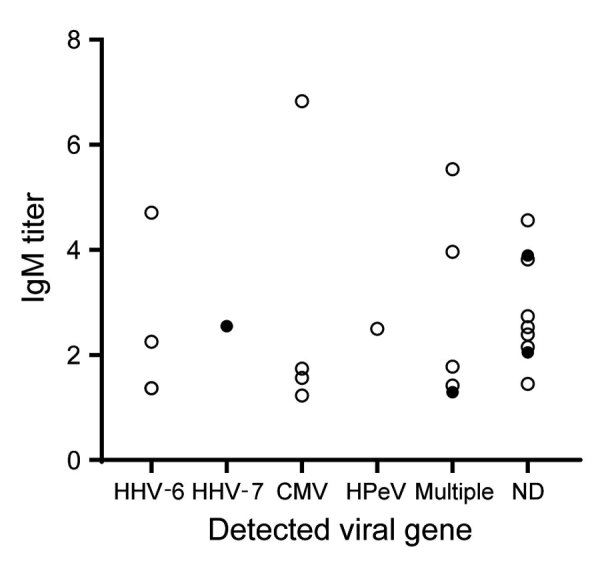
Measles-specific antibody titers and febrile exanthematous viral gene detection results in an analysis of suspected measles cases with discrepant measles-specific IgM and rRT-PCR test results, Japan. Figure represents 24 cases with measles-specific IgM-positive and rRT-PCR–negative results. White circles represent cases in children <1 year to 4 years of age with >1 doses of measles-containing vaccine; black circles represent cases in adults with unknown vaccination history. CMV, cytomegalovirus; HHV-6, human herpesvirus 6; HHV-7, human herpesvirus 7; HPeV, human parechovirus; multiple, multiple pathogens were detected; ND, not detected.

We detected viral nucleic acid other than measles virus in 15 cases, 13 in children and 2 in adults. Viruses detected from the children’s samples were HHV-6 (n = 8), CMV (n = 7), HPeV (n = 3), and EBV (n = 1). Among the adults, HHV-7 was identified in throat swab samples, and B19 was identified in a throat swab and serum sample. Multiple pathogens were detected in samples from 4 children and 1 adult. We noted no difference in the distribution of measles-specific IgM values between cases with and without viruses other than the measles virus detected (p = 0.318 by Mann–Whitney U test) (Figure 2).

## Discussion

Even after the declaration of measles elimination in 2015, measles outbreaks initiated by imported measles cases have occurred in Japan (*5*–*7*). Thus, accurate diagnosis of measles and continuous surveillance are required to maintain measles elimination status. In Japan, both the rRT-PCR and ELISA measles-specific IgM tests are recommended to confirm measles (*27*). Although rRT-PCR is the most reliable test to diagnose measles, its optimal time for specimen collection is limited to within 7 days after the symptom onset. Furthermore, measles-like symptoms can be caused by other viruses, such as rubella virus, B19, HHV-6, enterovirus, adenovirus, dengue fever, coxsackievirus, and several bacterial and rickettsia diseases (*11*,*28*). Consequently, unless a patient has had close contact with a confirmed measles case or measles is prevalent in the community, physicians might find it difficult to suspect measles only on the basis of symptoms and specimen collection at the optimal period could easily be missed. Therefore, a measles-specific ELISA IgM test is required to complement the short window for accurate diagnosis by rRT-PCR. The ELISA IgM test has a longer appropriate specimen collection period and is the reference standard for confirmation and surveillance of measles worldwide (*8*).

Results of rRT-PCR and ELISA measles-specific IgM usually agree, but diagnosis can be difficult for discrepant results, especially in cases of rRT-PCR–negative and IgM-positive results. We analyzed samples from 24 patients with suspected measles whose samples previously tested rRT-PCR–negative and IgM-positive during a 2018 outbreak in Okinawa Prefecture, Japan. To understand the cause of the discrepancy, we performed 2 additional tests on those patient samples, IgG avidity test and detection of viral nucleic acid other than measles virus. 

Measles-specific IgG avidity test results can provide useful information to distinguish between a recent infection or recent MCV vaccination, which are characterized by a low RAI, and past infection or past MCV vaccination, which are characterized by a high RAI (*15*,*29*). Among 20 suspected cases in children, 17 had low RAIs and 3 children (cases 18–20) showed equivocal or high RAIs. All 4 adult cases had high RAIs. The cases with low RAIs can be explained by recent MCV vaccination. Our results showed that in children who had received 1 dose of MCV, the RAI was correlated with the number of days after vaccination, consistent with results of a previous study (*15*). Suspected cases in children with equivocal or high RAIs had longer intervals between vaccination and sample collection compared with cases with low RAIs. One of the children (case 20) who had the longest interval between vaccination and sample collection tested positive for HHV-6 in a serum sample. Therefore, false-positive measles IgM could have been related to either an earlier MCV or an HHV-6 infection causing cross-reactivity, and we concluded that a measles infection was highly unlikely.

Four children (cases 1, 2, 5, and 9) had low RAIs and low measles-specific IgG antibody levels (<275 IU/L). All the samples from those cases were collected within 2 weeks after vaccination with MCV, which is before the body had time to produce a robust antibody response. We detected other viral pathogens in some specimens. In those cases, cross-reactivity with other viruses could have caused a false-positive measles IgM result. Although we could not establish the exact cause of the low RAIs with low IgG, those results ruled out a diagnosis of measles.

Four adult cases (cases 21–24) had high IgG titers and high RAIs shortly after the onset of the disease. Those results match previous studies that confirmed measles cases with either vaccination or natural infection show a low or undetectable IgM titer, a high RAI, and a high IgG titer in the early period after illness onset (*30*). Among the 4 adult cases, we detected febrile exanthematous viruses other than measles from the acute phase specimens of 2 cases (cases 21 and 24). The subtle increase of measles IgM titer for those cases was possibly caused by a cross-reaction with other viruses rather than measles infection. Completely ruling out measles on the basis of results of avidity testing and tests for other pathogens is difficult when the measles vaccination and previous measles disease history are unknown. Therefore, when in doubt, clinicians should treat indeterminate cases as positive in terms of the public health response.

Vaccine-associated measles cases can be detected using rRT-PCR if symptoms occur within 2 weeks after vaccination and specimens are collected within 7 days from symptom onset (*7*). One limitation of this study is that rRT-PCR possibly did not detect the vaccine strain because most children with an elevated measles-specific IgM titer had not received MCV within the past 2 weeks. However, even though the vaccine strain was not detected, taking the RAI result and immunization date information into consideration, we can infer that the symptoms were caused by the vaccine strain. Another limitation of this study is that we only conducted nucleic acid testing for a few viral pathogens that cause febrile exanthemata; thus, the symptoms might have been attributable to other viruses or bacteria that cause similar symptoms. Next-generation sequencing could help to identify the causal pathogen of febrile exanthemata. In addition, results of this study indicate a complete measles diagnosis would require additional testing, such as paired IgG and neutralizing antibody tests. However, collecting convalescent serum samples and conducting neutralizing antibody tests would require more time and would not be suitable when rapid determination of measles cases is needed in an outbreak setting. Therefore, we measured IgG levels as part of the avidity test instead of performing paired IgG testing.

During outbreaks, field staff are extremely busy investigating the source of infection and close contacts. Accurately identifying measles cases is vital for a rapid and accurate outbreak response and maintaining measles elimination status in Japan. Therefore, for suspected measles cases, collecting specimens at the appropriate time and collecting accurate vaccination and past infection history are crucial. When a definitive diagnosis still cannot be made, conducting IgG avidity testing and testing for febrile exanthematous pathogens other than measles virus can help clarify the diagnosis.

In conclusion, 2 diagnostic tests, rRT-PCR and measles-specific IgM, are used in Japan to maintain measles elimination status. Because 2 tests are conducted, test results sometimes differ, making definitive diagnosis difficult. Our results indicate that conducting measles IgG avidity testing and PCR testing for other febrile exanthematous viruses and collecting a detailed history of measles vaccination and measles history can reduce the difficulty of making a final diagnosis in most cases with discrepant rRT-PCR and IgM results.

Appendix Additional information on analysis of suspected measles cases with discrepant measles-specific IgM and rRT-PCR test results, Japan.
