# Supplementary material for: Analysis of Suspected Measles Cases with Discrepant Measles-Specific IgM and rRT-PCR Test Results, Japan
Source: Emerg Infect Dis. 2024 May;30(5):926–33. doi: 10.3201/eid3005.231757 (PMC11060445; doi:10.3201/eid3005.231757)
Supplement: Appendix — Additional information on analysis of suspected measles cases with discrepant measles-specific IgM and rRT-PCR test results, Japan. [file 23-1757-Techapp-s1.pdf]

Article DOI: https://doi.org/10.3201/eid3005.231757

EID cannot ensure accessibility for supplementary materials supplied by authors. Readers who have difficulty accessing supplementary content should contact the authors for assistance.

Analysis of Suspected Measles Cases with Discrepant Measles-Specific IgM and rRT-PCR Test Results, Japan

Appendix

Appendix Table. Primer and probe sequences used to detect febrile exanthematous viruses other than measles virus using real-time reverse transcription PCR or PCR*

Virus	Primer/probe	Sequence (5′→3′)	Reporter-quencher	Ref. no.
Rubella virus	NS(32–54)Fwd	CCTAHYCCCATGGAGAAACTCCT	FAM-MGB	(*1*)
	NS(143–160)Rev	AACATCGCGCACTTCCCA		
	NS(93–106)Probe	CCGTCGGCAGTTGG		
Enterovirus	Forward primer	CCCTGAATGCGGCTAATCC	FAM-BHQ1	(*2*)
	Reverse primer	ATTGTCACCATAAGCAGCCA		
	Probe	AACCGACTACTTTGGGTGTCCGTGTTTC		
Human parechovirus	ParechoF31	CTGGGGCCAAAAGCCA	FAM-MGB	(*3*)
	K30	GGTACCTTCTGGGCATCCTTC		
	HPeV-WT-MGB	AAACACTAGTTGTAWGGCCC		
Human herpesvirus 6	TAQ6E	CAAAGCCAAATTATCCAGAGCG	FAM-MGB	(*4*,*5*)
	TAQ6B	CGCTAGGTTGAGAATGATCGA		
	HHV6 probe	CACCAGACGTCACACCCGAAGGAAT		
Human herpesvirus 7	TAQ7F	ATGTACCAATACGGTCCCACTTG	FAM-MGB	(*6*)
	TAQ7R	AGAGCTTGCGTTGTGCATGTT		
	HHV7 probe	CACGGCAATAACTCTAG		
Parvovirus B19	Parvo B19_fw	AATGCAGATGCCCTCCA	FAM-MGB	(*7*)
	Parvo B19_rev	ATGATTCTCCTGAACTGGT		
	Parvo B19 Taq_probe	AACASTGARACCCCGCGCTCTAGTAC		
Epstein-Barr virus	Forward primer	CTTAGAATGGTGGCCGGGCTGTAAAAT	NA	(*8*)
	Reverse primer	ATCCAGTACGTCTTTGTGGAGCCCAAG	NA	
Cytomegalovirus	Forward primer	GCGCGTACCGTTGAAAGAAAAGCATAA	NA	(*8*)
	Reverse primer	TGGGCACTCGGGTCTTCATCTCTTTAC	NA	
Adenovirus	Forward primer	TTCCCCATGGCNCACAAYAC	NA	(*9*)
	Reverse primer	TGCCKRCTCATRGGCTGRAAGTT	NA	

*BHQ1, black hole quencher 1; FAM, carboxyfluorescein; Fwd, forward; HHV, human herpesvirus; MGB, minor groove binder; NA, not applicable; Ref., reference; Rev, reverse.

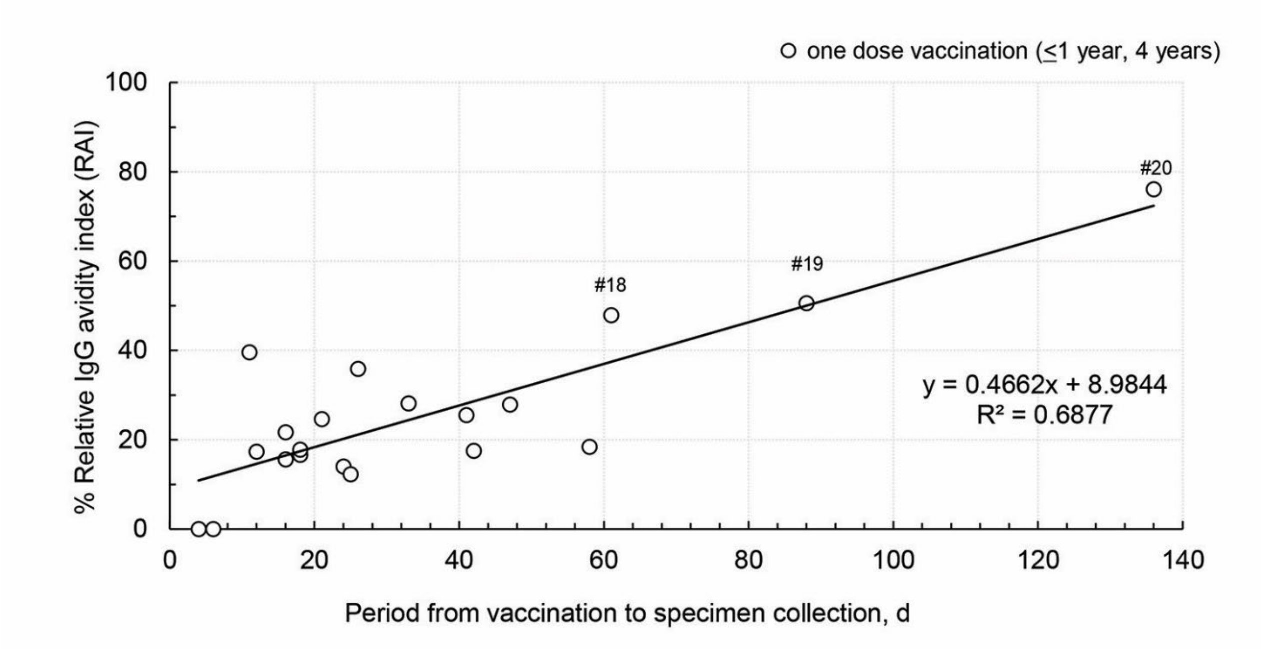

Appendix Figure. Correlation between the relative measles IgG avidity index and the interval from vaccination to specimen collection in 20 children who had 1 dose of measles vaccine, Japan. Nos. 18–20 correlate to patient numbers in Table 2. IgG, immunoglobulin G; RAI, relative avidity index.

References

1. Okamoto K, Fujii K, Komase K. Development of a novel TaqMan real-time PCR assay for detecting rubella virus RNA. J Virol Methods. 2010;168:267–71. PubMed https://doi.org/10.1016/j.jviromet.2010.05.016

2. Verstrepen WA, Kuhn S, Kockx MM, Van De Vyvere ME, Mertens AH. Rapid detection of enterovirus RNA in cerebrospinal fluid specimens with a novel single-tube real-time reverse transcription-PCR assay. J Clin Microbiol. 2001;39:4093–6. PubMed https://doi.org/10.1128/JCM.39.11.4093-4096.2001

3. Benschop K, Molenkamp R, van der Ham A, Wolthers K, Beld M. Rapid detection of human parechoviruses in clinical samples by real-time PCR. J Clin Virol. 2008;41:69–74. PubMed https://doi.org/10.1016/j.jcv.2007.10.004

4. Locatelli G, Santoro F, Veglia F, Gobbi A, Lusso P, Malnati MS. Real-time quantitative PCR for human herpesvirus 6 DNA. J Clin Microbiol. 2000;38:4042–8. PubMed https://doi.org/10.1128/JCM.38.11.4042-4048.2000

5. Ogawa H, Suzutani T, Baba Y, Koyano S, Nozawa N, Ishibashi K, et al. Etiology of severe sensorineural hearing loss in children: independent impact of congenital cytomegalovirus infection and GJB2 mutations. J Infect Dis. 2007;195:782–8. PubMed https://doi.org/10.1086/511981

6. Fernandez C, Boutolleau D, Manichanh C, Mangeney N, Agut H, Gautheret-Dejean A. Quantitation of HHV-7 genome by real-time polymerase chain reaction assay using MGB probe technology. J Virol Methods. 2002;106:11–6. PubMed https://doi.org/10.1016/S0166-0934(02)00131-3

7. Takao S, Shigemoto N, Shimazu Y, Tanizawa Y, Fukuda S, Matsuo T. Detection of exanthematic viruses using a TaqMan real-time PCR assay panel in patients with clinically diagnosed or suspected measles. Jpn J Infect Dis. 2012;65:444–8. PubMed https://doi.org/10.7883/yoken.65.444

8. Tanaka T, Kogawa K, Sasa H, Nonoyama S, Furuya K, Sato K. Rapid and simultaneous detection of 6 types of human herpes virus (herpes simplex virus, varicella-zoster virus, Epstein-Barr virus, cytomegalovirus, human herpes virus 6A/B, and human herpes virus 7) by multiplex PCR assay. Biomed Res. 2009;30:279–85. PubMed https://doi.org/10.2220/biomedres.30.279

9. Miura-Ochiai R, Shimada Y, Konno T, Yamazaki S, Aoki K, Ohno S, et al. Quantitative detection and rapid identification of human adenoviruses. J Clin Microbiol. 2007;45:958–67. PubMed https://doi.org/10.1128/JCM.01603-06
